# The modulatory role of second language proficiency on performance monitoring: evidence from a saccadic countermanding task in high and low proficient bilinguals

**DOI:** 10.3389/fpsyg.2014.01481

**Published:** 2015-01-05

**Authors:** Niharika Singh, Ramesh K. Mishra

**Affiliations:** ^1^Centre of Behavioural and Cognitive Sciences, University of AllahabadAllahabad, India; ^2^Centre of Neural and Cognitive Sciences, University of HyderabadHyderabad, India

**Keywords:** bilingualism, redirect task, inhibition, performance monitoring, saccades

## Abstract

We compared Hindi-English bilinguals differing in their L2 proficiency on a saccadic countermanding task which taps inhibitory control as well as monitoring. We particularly explored whether response inhibition and performance monitoring within the oculomotor domain are affected by language proficiency in bilinguals. There were two different oculomotor redirect tasks: Visually Guided Redirect (VGR) task (Experiment1) and Memory Guided Redirect (MGR) task (Experiment 2). In the redirect task, typically a target is presented and the subject is required to make a saccade (no-step trials), unless a new target appears on a different location after some delay from the first target onset (step trials). On such trials participants are required to inhibit and cancel the saccade to the first target and programme a saccade to the new target. Using trial switch reaction time (TSRT), the time taken to inhibit the initiated saccade to the first target as a measure of response inhibition and post-step slowing as a measure of performance monitoring. The results showed the high proficient bilinguals displayed more post-step slowing on the no-step trials as compared to the low proficient bilinguals for both VGR and MGR versions of the task. Secondly, both the high and low proficient bilinguals exhibited comparable TSRT in both VGR and MGR task, showing no modulatory effects of language proficiency on the response inhibition. These results suggest that language proficiency may have an effect on performance monitoring, but not the inhibitory control *per se*. Thus, we infer that higher proficiency may lead to superior cognitive flexibility and an ability to adjust behavior that facilitates the attainment of the cognitive goal. These findings are in consonance with other current studies that suggest a top-down effect of bilingualism on action control systems.

## Introduction

Bilingualism has been known to positively influence several cognitive skills such as conflict resolution (Green, 1998; Bialystok, 1999; Bialystok et al., 2004, 2005, 2008; Bialystok and Martin, 2004); selective attention (Colzato et al., 2008; Friesen et al., 2014), monitoring (Costa et al., 2008, 2009; Singh and Mishra, 2013); anticipation (Kovács and Mehler, 2009); and top down control (Hernández et al., 2012) etc. The unique processing demands placed on bilinguals in managing the two languages make them cognitively more flexible compared to monolinguals. Constantly shifting between two languages, selecting the context appropriate language and suppressing the context inappropriate language (see Bialystok and Craik, 2010) leads to the enhancement and strengthening of general purpose executive functions in different non-linguistic domains in bilinguals. Bilinguals have been shown to outperform monolinguals on a wide variety of tasks requiring attentional and executive control such as such, as the Stroop task (Hernández et al., 2010; Singh and Mishra, 2012, 2013), the Simon task (Bialystok et al., 2004; Martin-Rhee and Bialystok, 2008; Salvatierra and Rosselli, 2011), Dimension card sorting task (Bialystok, 1999; Bialystok and Martin, 2004; Carlson and Meltzoff, 2008), the Attentional network task (Costa et al., 2008, 2009), and anti-saccade task (Bialystok et al., 2006). Despite numerous evidence showing bilingual cognitive advantage, there is little unanimity on the aspect of bilingualism that contributes to enhanced executive control (Paap and Greenberg, 2013). Theorists seem to be divided between a reactive inhibitory control account (Green, 1998) and accounts that support a more top-down goal directed performance in bilinguals (Colzato et al., 2008). Further, many studies till date have compared bilinguals with monolinguals that may not be ecologically valid (Kroll and Bialystok, 2013). In the present study, we examined how second language proficiency affects performance monitoring and response inhibition in bilinguals in the oculomotor domain.

An important issue concerning how bilingualism modulates cognitive control is to explain if bilinguals inhibit/suppress a task irrelevant response or they exercise constant monitoring of their behavior using top down control. Hilchey and Klein (2011) in their review suggested that bilingualism may enhance general executive control processes but not inhibitory control *per se*. An important aspect of executive control is to monitor one's behavior while keeping current goals for optimal performance (Festman and Münte, 2012). Performance monitoring helps to remain alert toward errors and maintain constant vigilance during the task (Botvinick et al., 1999). While previous studies have looked at monitoring in the context of uncertainty (Costa et al., 2009; Singh and Mishra, 2013), it is still not clear, if bilinguals show enhanced ability in avoiding errors and maintaining task goals. Costa et al. (2009) compared bilinguals and monolinguals on the flanker task under high and low monitoring contexts. The monitoring context was manipulated by varying the proportion of congruent and incongruent trials such that when the number of congruent and incongruent trials were equal or almost equal in proportion (i.e., 50 and 75% congruence proportion), it constituted high monitoring context while in low monitoring context only one type of trials were presented (i.e., 98% congruent or 98% incongruent trials). The results showed that bilinguals were overall faster than the monolinguals only in the high monitoring context, suggesting that bilinguals use their executive control to a greater extent when the monitoring demands are higher. Costa et al. (2009) proposed that bilingual advantage on executive functions is an outcome of specific cognitive components which are recruited as per the communicative demands of the bilinguals. Bilinguals keep track of the code shifts on the part of the interlocutor so that conversation flows smoothly, which calls for monitoring. This requirement of constant monitoring, results in the enhancement of the monitoring system in bilinguals.

Recently, many other studies also have observed that bilinguals and monolinguals may not differ from one another on tasks that call for conflict resolution or inhibitory control (Paap and Greenberg, 2013; see also Valian, 2014). Colzato et al. (2008) did not find any bilingual advantage in the stop signal task where participants had to inhibit the motor response on some trials. Furthermore, others have not observed any advantage for bilinguals on tasks measuring conflict resolution or inhibitory control (Kousaie and Phillips, 2011; Paap and Greenberg, 2013; Duñabeitia et al., 2014). Even with inhibition, it appears that bilinguals may do better at the response selection level, but not during response inhibition (Luk et al., 2010). For instance, bilinguals have been found to show executive control advantage on tasks that require suppression of interference, but not when tested on tasks requiring an inhibition of pre-potent responses (Martin-Rhee and Bialystok, 2008; Esposito et al., 2013).

In this study, we explored how bilingual language proficiency may influence oculomotor control in a task which requires performance monitoring. Flexible behavioral adjustment is an essential aspect of the executive control system. It requires continuous assessment of actions and their consequences in order to evaluate if they are in compliance with our internal cognitive goals. For example, in the context of bilinguals, changing demands of conversational settings may call for an additional updating in planning goals. Performance monitoring can be implemented by detection of error (Rabbitt, 1966) or by monitoring competing responses (Botvinick et al., 1999) or by both (Ullsperger and Von Cramon, 2001). Thus, performance monitoring serves as an adaptive mechanism so that once the conflict or error has been detected; a behavioral adjustment can take place to maximize performance for the attainment of the cognitive goal (see Botvinick et al., 2001). Performance monitoring is often observed as slowing of responses on trials that follow an erroneous performance on the previous trials or when it is preceded by a trial that involves conflict. However, it has always remained debatable whether error or conflict detection serves as a signal for performance monitoring (Ullsperger and Von Cramon, 2001). There are mixed evidences; however the majority of the studies indicate that it is the presence of a conflict that mediates performance monitoring. Festman and Münte (2012) compared two groups of highly proficient bilinguals differing in language control ability (late switchers and non-switchers) on the Wisconsin Card Sorting Test and the Flanker task. The non-switchers performed better on the Flanker task indicating superior conflict resolution. Further, ERP data showed that non-switchers had a smaller error related negativity, which indicates a superior monitoring system. This study shows that language control abilities influence the monitoring system depending on the frequency of unintentional switching behavior. Brain imaging evidence also suggests that bilinguals may deal with sustained and transient language control differently, depending on the linguistic situation i.e., switch or no-switch (Wang et al., 2009). Therefore, bilingualism boosts the ability to constantly monitor situations that often include conflict. Very few studies have explored if bilingualism influences monitoring of performance during tasks that do not call for active suppression of the interference, but where one has to constantly take alternative decisions and sometimes cancel the already prepared decision, as it is the case with the stop signal paradigm (Logan and Cowan, 1984). Although Costa et al. (2009) had explored monitoring in bilinguals; they had employed a Flanker task which calls for active conflict resolution. Therefore, it is important to examine performance monitoring in a task that does not have conflict as such but which calls for constant changes to goal directed action depending on trial demands. Below we explain the task used in this study where participants did not have to manage conflict as such but where they had to cancel planned action depending on the nature of trials.

The adaptive control hypothesis (Green and Abutalebi, 2013) proposes that the language control abilities/mechanisms in bilinguals are dynamic and get adapted as per the communicative demands and interactional context. Recent evidence suggests that L1/L2 proficiency has a modulatory effect on the bilingual advantages. Singh and Mishra (2012, 2013) observed the effects of second language proficiency on conflict resolution, as well as, monitoring in high proficient Hindi-English bilinguals. High and low proficient Hindi-English bilinguals were compared on a saccadic Stroop task under different monitoring situations. Higher L2 proficiency was linked not only to the superior performance on the conflict resolution, but also with overall faster response latencies indexing superior monitoring in an oculomotor domain. This may suggest that a monitoring account may not be completely independent of an inhibitory control account. Therefore, it is important to further examine the interaction between monitoring and inhibitory control in bilinguals with different proficiency levels. Tao et al. (2011) examined early and late bilinguals on the lateralized ANT task and found that late, but balanced bilinguals exhibited better conflict resolution abilities while early bilinguals showed a global RT advantage suggesting an efficient monitoring system. Tse and Altarriba (2012) using the Stroop task had found goal maintenance and conflict resolution was associated with both L1/L2 proficiency. Further adding to the evidence, Coderre et al. (2012) also have observed a robust evidence of superior control abilities in high proficient bilinguals as compared to the monolinguals while testing them on the Stroop task.

Very few studies have examined how bilingualism influences performance in the oculomotor domain since most studies till date have examined tasks with manual response, given the fact that cognitive and neural mechanisms sub-serving manual and ocular response system may differ and further the saccadic response system more directly manifest attentional engagement and control (Hoffman and Subramaniam, 1995). Bialystok et al. (2006) compared bilinguals and monolinguals on an anti-saccade task which measures response inhibition. In the pro-saccade trials participants were asked to look toward the target while in the anti-saccade trials participants were required to inhibit reflexive saccades toward the target and look in the opposite direction. The authors found no bilingual advantage and the results showed a comparable performance on the anti-saccade trials by both the groups. Others have examined how manual and oculomotor inhibition is linked to parallel language activation in bilinguals. Mercier et al. (2014) examined how oculomotor inhibitory control measured with anti-saccade task could explain the magnitude of cross-language activation in bilinguals in a visual world task. The authors observed that increased oculomotor inhibitory control was linked to lesser cross-language competition. This suggests that performance on oculomotor tasks can predict language processing. On the other hand, it is also possible to state that certain characteristics of bilingual language use can enhance oculomotor control. Blumenfeld and Marian (2011) had found that inhibitory control on a non-linguistic Stroop task predicted resolution of parallel language competition in bilinguals but not in monolinguals during spoken word processing. Similarly, language proficiency has been shown to correlate with performance on the Stroop task where manual responses have been collected (Marian et al., 2013). Therefore, bilingualism should have an effect on oculomotor control in different tasks.

In order to investigate performance monitoring and response inhibition we used the oculomotor redirect task. Given the very close relationship between attention and saccade programming (Hoffman and Subramaniam, 1995) it is likely that eye movement measures are sensitive to the cognitive influence of bilingualism. The oculomotor redirect task, a variation of the stop-signal task, is especially designed to test oculomotor inhibitory control (Kapoor and Murthy, 2008; Joti et al., 2007). Previous studies have shown that this task can also reveal error related awareness in participants (Endrass et al., 2005). Specific brain areas like the anterior cingulate cortex have been found to be active during performance monitoring in this task (Ito et al., 2003). The saccade countermanding task has been used extensively to study response adjustments during action control (Schall et al., 2002). In this task, typically a target is shown to which the participants must make a saccade (no-step trials), unless a new target appears at another location after after some delay from the first target onset (step-trials). On such trials participants are required to inhibit and cancel the saccade to the first target and shift their gaze to the new target (step-target) location. This voluntary redirecting of gaze to a new location requires stopping of the already planned saccade and is influenced by the delay between the first and the second target. If the delay is long enough, then subjects often end up making a saccade to the original target erroneously, but if the delay is short, then subjects may successfully cancel the saccade to the original target and plan another saccade to the new target. The time taken to inhibit the initiated saccade to the first target is called as trial switch reaction time (TSRT). Any failure to inhibit the saccade leads to an erroneous saccade to the first leading to non-canceled saccades. The TSRT along with the number of non-canceled saccades give a measure of response inhibition. It has been assumed that stopping requires some form of active inhibition of a response. While the stop-signal reaction time (SSRT) has been a very popular measure of inhibition, reflecting a dynamic competition between the go and stop processes (Logan and Cowan, 1984); recently alternative accounts of the mechanisms involved in the stop signal paradigm have been proposed. Salinas and Stanford (2013) offer a perceptual detection threshold account of the stop signal task and have argued that such an account may not require assumptions of inhibition. This model focuses on the processing speed of the subject to process perceptual cues and stop action (Salinas and Stanford, 2013). Thus, the detectability of the stop-signal can affect the action control processes in such tasks. Salinas and Stanford (2013) propose a tachometric curve that captures the correlation between SSRT and timing of perceptual detection processes. Viewed from this angle, inhibition is not necessary as a mechanism in the stop signal task and one can account for the SSRTs observed by looking at the effectiveness of the perceptual detection processes. While this model captures some important cognitive and perceptual aspects of the countermanding task, it has been suggested that this model does not improve on existing race models as such (Bisset, 2013). Be it as it may, it is not our aim here to examine the strengths and weakness of different models that capture control mechanisms in the stop-signal task, but to see how this task can be used to capture cognitive influences of bilingualism in a saccade task.

The probability of making a correct saccade to the step target on the step trials depends upon the race between two independent processes: stop and go (Logan and Cowan, 1984), such that whichever finishes first is executed first. However, successful performance on the redirect task does not only depend upon faster execution of stop processes. Rather, it involves monitoring of both go and stop processes dynamically. This is necessary to bring in an adjustment in the response strategies to efficiently switch between the go and stop tasks which place conflicting demands. The adjustment in the response strategies is often observed as a trade-off between the go and the stop processes as speeded responses on the go trials may have hampering effect on the probability of inhibiting response on the stop trials. It has been found that slowing of response on the go trials is common when they follow stop-signal trials to minimize the probability of making errors on stop trials. The response to those go trials that follow stop signal trials are slower, and this phenomena is called as the “post stop slowing” (Verbruggen and Logan, 2008; Bissett and Logan, 2011; Stuphorn and Emeric, 2012). Post-stop slowing becomes more prominent when it occurs after successful and unsuccessful (error) inhibition on the trials with stop-signals. Such post-stop slowing reflects performance monitoring. Compared to tasks used in earlier studies of oculomotor control in bilinguals (Singh and Mishra, 2012, 2013), this task requires a constant monitoring of the situation which changes dynamically. This task emphasizes one's ability to immediately stop a planned action at advanced stages of execution rather than just inhibiting a response on a regular basis to certain type of trials (as is the case with the Stroop or Simon type tasks). This is very close to how bilinguals manage the correct language code during conversations, since code shifts can be dynamic. The uncertainty of the task utilizes the performance monitoring system as well as calls for cognitive flexibility.

We administered two different versions of the redirect task: the visually guided redirect task (Experiment 1) and the memory guided redirect task (Experiment 2) on high and low proficient bilinguals. We tested following predictions to assess performance monitoring and response inhibition in these bilinguals:

1. If language proficiency modulates performance monitoring then the high proficient bilinguals, in general should show more post-step slowing on the no-step trials as compared to the low proficient bilinguals.
2. If higher L2 proficiency modulates all forms of inhibition, then we expect high proficient bilinguals to show smaller TSRT and smaller number of non-canceled saccades as compared to the low proficient bilinguals. However, if L2 proficiency only influences interference suppression and not response inhibition then we expect that the high and low proficient bilinguals will not differ on the measure of response inhibition, thus showing comparable TSRT and number of non-canceled saccades.

## Experiment 1

### Methods

#### Participants

Forty seven Hindi-English bilinguals took part in the study. All bilinguals were native speakers of Hindi studying at the University of Allahabad, India and had acquired English as L2 at school. In India, there are two types of school; one, where the medium of instruction and education is in the native language (i.e., Hindi in this case), and second, where the medium is English. In the Hindi-medium schools, all the courses are in Hindi and English is taught as second language. In the English-medium schools, all the courses are taught in English and Hindi is taught as one of the languages. In the present study, the language used as the medium of instruction (i.e., Hindi or English) at school for education was used as a preliminary criterion to divide the bilinguals into two proficiency groups; such that bilinguals who went to Hindi-medium school were classified as low proficient bilinguals and those who went to English-medium school as high proficient bilinguals, with regard to their proficiency in English. Based on this, participants were assigned to high (*N* = 22) and low L2 proficiency group (*N* = 25). To incorporate group differences, the participants in two groups were further administered the language background questionnaire, as well as the LexTALE task. However, it is important to note that by the time participants took part in the study, they were students in the University and had already three to five years of post-school education in English. The language background questionnaire required participants to report the number of languages known by them. All the participants reported Hindi as their L1 and English as L2 (except for two who reported to also know “Bhojpuri,” which is a Hindi dialect, in addition to Hindi and English). The participants also reported their formal age of acquisition of L1 and L2, language usage pattern of L1 and L2 (see **Table 2**). The language questionnaire also included a section on self-rating of proficiencies on reading, writing, speaking, and listening in both languages. The participants rated their proficiency on a 5-point Likert scale (where 1 represented *poor*, while 5 presented *excellent proficiency*). The high proficient bilinguals were significantly higher in reading, writing, speaking and listening proficiencies in L2 as compared to the low proficient bilinguals (see Table 1).

**Table 1:** **Self-ratings for reading, writing, speaking, and listening in L1 and L2**.

	**Speaking**		**Listening**		**Reading**		**Writing**	
	**L1**	**L2**	**L1**	**L2**	**L1**	**L2**	**L1**	**L2**
HPB	4.8 (0.39)	4.0 (0.69)	4.8 (0.35)	4.2 (0.35)	4.6 (0.58)	4.3 (0.65)	4.4 (0.79)	4.0 (0.69)
LPB	4.8 (0.30)	2.2 (0.96)	4.6 (0.47)	2.9 (0.47)	4.8 (0.40)	3.3 (1.1)	4.5 (0.50)	3.0 (0.97)
		^**^		^**^		^**^		^**^

*Standard deviations are given in parentheses*.

*L1, Hindi; L2, English. High and low proficiency based on L2 proficiency*.

** *p < 0.01*.

Apart from self-rating, some more objective measures of proficiency were also conducted. These included reading comprehension tests, both in L1 and L2, and LexTALE task (Lemhöfer and Broersma, 2012). Comprehension passage scores showed that high proficient bilinguals significantly scored more on the English (L2) comprehension passage test as compared to the low proficient bilinguals while the scores of two groups were comparable on the Hindi (L1) comprehension passage test (see Table 2). LexTALE is a test of English vocabulary knowledge which indicates English proficiency (Lemhöfer and Broersma, 2012). This test requires the participants to judge strings of letters as words or non-words in English. This task has been previously used as a proficiency measure in bilingualism research (Khare et al., 2013). The results of LexTALE in the present study showed that high proficient bilinguals showed significantly higher score percentage as compared to the low proficient bilinguals (see Table 2).

**Table 2:** **Demographic details, non-verbal IQ, LexTALE, and comprehension passage scores of High-proficient bilinguals (HPB) and low proficient bilinguals (LBP)**.

	**HPB**	**LPB**
Mean age	21.4 (5.2)	20.5 (2.3)
Mean formal age of L1 acquisition (years)	3.6 (0.77)	4.1 (0.68)
Mean formal age of L2 acquisition (years)	3.9 (0.81)	4.5 (8.86)
Hours of conversation in L1	2.5 (1.0)	4.3 (1.1)^*^
Hours of conversation in L2	5.1 (1.7)	1.18 (0.78)^*^
Non-verbal IQ	52.9 (3.7)	51.5 (3.5)
Socio-economic status	2.3 (0.77)	2.1 (0.55)
Mean score in L1 comprehension (out of 5)	4.5 (0.67)	4.3 (0.71)
Mean score in L2 comprehension (out of 5)	4.6 (0.47)	2.0 (1.35)^**^
LexTALE score percentage	82.9 (8.1)	56.4 (7.8)^**^

*Standard deviations are given in parentheses*.

*L1, Hindi; L2, English. Non-verbal IQ: Ravens Progressive Matrices, out of 60. See text for explanation*.

* p < 0.05,

** *p < 0.01*.

The two groups were matched on non-verbal IQ (Raven's Progressive matrices) and socio-economic status. To assess the socio-economic status participants were required to indicate, on a 3-point scale, to which socioeconomic class they belonged (1 for “lower middle class,” 2 for “middle class,” and 3 for “upper middle class”) (see Table 2).

### Ethics statement

The study was approved by the ethics committee of the Allahabad University. All the participants gave their informed consent before taking part in the study and were paid for their participation. The researcher who collected data also signed each consent form. They were also told that privacy as per law would be maintained with regard to their data.

#### Apparatus and stimuli

Eye position was monitored by SMI High speed eye-tracking system (Sensomotoric Instruments, Teltow), with sampling rate of 1250 Hz. The stimuli were delivered through PRESENTATION software (Neurobehavioral systems) on a 17 ” colored monitor with a resolution of 1024 × 768. The participants were comfortably seated at a distance of 75 cm from the computer monitor with their head fixed on a chin rest. The fixation was a black square while No step and step targets were red and green in color respectively each extending 1° × 1°. Stimuli were presented on a white colored background.

#### Procedure

**Figure 1:**
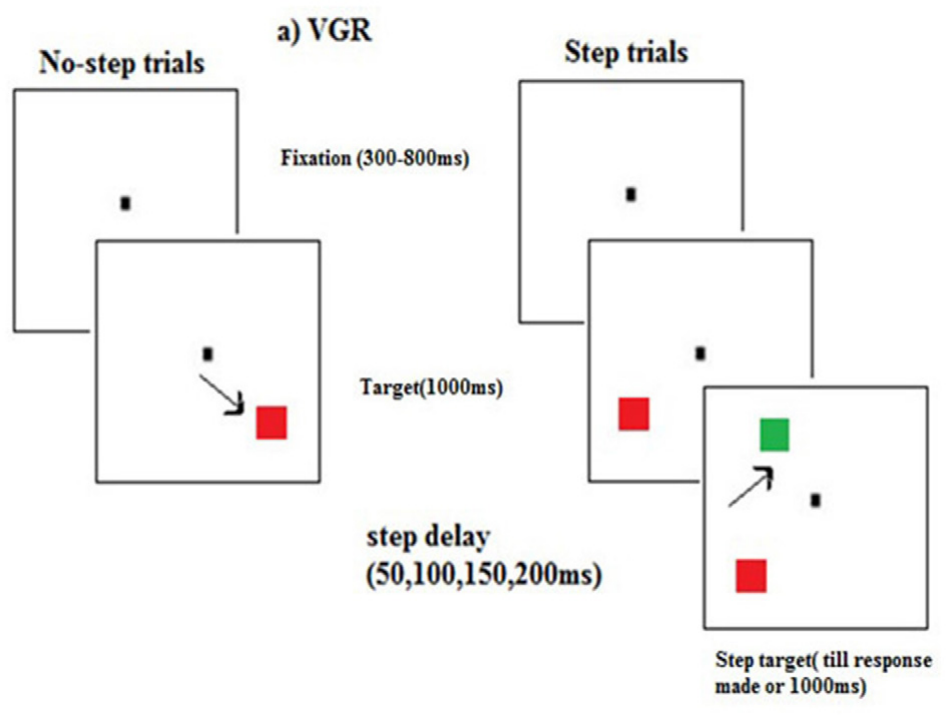
**A sample trial sequence for step and no-step trials in the visually guided redirect (VGR) saccade task**. In no-step trials participants were instructed to look at the red square (target). In the step trials participants were required to look at the green square (step target) which appeared after some delay (50, 100, 150, and 200 ms) from the onset of the red square.

The experiment began with a calibration process where participants were asked to look at a target that appeared at random locations on the screen. The calibration process was automatic. The participants performed the oculomotor redirect task in which 60% of trials were no-step trials. On these trials participants had to look at a central fixation which appeared at the center of the screen. After a variable period of delay ranging from 300 to 800 ms a red color square target appeared at any of the six possible locations on a circumference of an imaginary circle with aradius10°. The target remained on the screen for 1000 ms. On these no-step trials participants were instructed to make a saccade to the target as quickly as possible. However, on 40% of trials, the red colored target was followed by a green color square (step target). These were called as step trials. The step target was presented at four different target step delays (TSD = 50, 100, 150, and 200 ms). TSD is the duration gap between the appearances of step target after the onset of first target. The step target could appear on any of the remaining 5 locations of the imaginary circle. On these trials participants were instructed to cancel the saccade to the red square and instead make a saccade to the step target. The maximum duration of responding was 1000 ms but the trial ended soon after the participant has made a correct saccade to the step target or after a delay of 1000 ms which was maximum duration of responding. In case of a time out or incorrect saccades, a tone of 400 Hz was presented as an alert (Figure 1). The experiment had a total of 600 trials, out of which 240 were step trials with 60 trials presented at each TSD).

#### Data analysis

Eye tracking data were analyzed using the BeGaze analysis software (Sensomotoric Instruments, Teltow) and Python, an open source programming platform (www.python.org). A saccade was defined as a movement of the eye more than 30°/s, following a velocity criterion from its present position in any direction. The AOI (area of interest), for calculation of saccades and their latencies, was about the same size as the target.

### Results and discussion

#### Number of non-canceled step saccades

On step trials when participants failed to inhibit the saccade to the first target and made an erroneous saccade toward it, it was counted as a non-canceled step saccade. We compared whether the two groups differed on successfully inhibiting saccades to the first target on the step trials, by calculating the number of non-canceled saccades for each target step delay (TSD). The number of non-canceled saccades were subjected to a repeated measure ANOVA, with TSD (50, 100, 150, and 200 ms) as a within subjects' factor and group (high and low proficient bilinguals) as between subjects' factor. The main effect of group was significant, *F*_(1, 45)_ = 6.9, *p* = 0.03, showing a higher number of non-canceled saccades for the low proficient group (39.4, *SE* = 2.0) than the high proficient group (32.9, *SE* = 2.1). The main effect of TSD was significant, *F*_(3, 135)_ = 57.7, *p* = 0.001; revealing a significant gradual increase in the number of non-canceled saccades as the TSD increased (see Table 3). The interaction between TSD and group was not significant, *F*_(3, 135)_ = 1.3, *p* = 0.27.

**Table 3:** **Mean number of non-canceled saccades per target step delay (TSD) for the groups**.

	**Number of non-canceled saccades (VGR)**	
**TSD (ms)**	**High proficient bilinguals**	**Low proficient bilinguals**
50	25.2 (2.4)	32.1 (2.2)
100	30.8 (2.4)	39.1 (2.2)
150	35.6 (2.4)	39.8 (2.3)
200	40.1 (2.1)	46.1 (2.0)

*Standard deviations are given in parentheses*.

#### Trial switch reaction time (TSRT)

**Figure 2:**
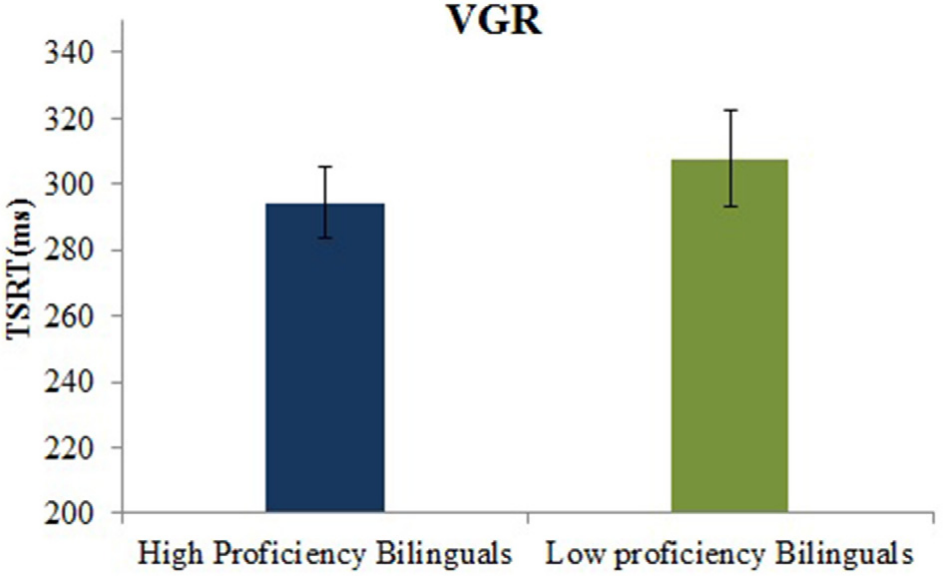
**Mean TSRT (ms) for high and low proficient bilinguals on the VGR task**.

TSRT is the time taken to cancel the first saccade and programme a new saccade toward the second target on the step trials. It is not directly available from the behavioral data and is estimated by using the no-step trial RT distribution and the probability of making error as a function of TSD (see Kapoor and Murthy, 2008 for details). The *t*-test revealed no significant difference, *t*_(45)_ = −0.71, *p* = 0.47, in the TSRT for high proficient bilinguals (294.1 ms, *SD* = 50.8) and the low proficient bilinguals (307 ms, *SD* = 73.4) (See Figure 2).

#### Post-error and -post conflict slowing (performance monitoring)

To see the effect of performance monitoring we calculated saccade latency for the no-step trials immediately following the step trials, for both the groups. Saccade latencies less than 80 ms (anticipatory) and greater than 1000 ms were excluded from the analysis. Depending on the trial type that preceded the no-step trial, three conditions were created. No-step trial was labeled as a post-conflict trial when a no-step trial was preceded by a correct step trial. When a no-step trial followed a non-canceled (error) step trial then it was labeled as a post-error trial, whereas when a no-step trial followed a no-step trial then it was called as a post-no-step trial. A repeated measure ANOVA was conducted with no-step trial type (post-conflict, post-error, and post-no-step) as a within subjects' factor and group (high and low proficiency) as a between subjects' factor.

**Figure 3:**
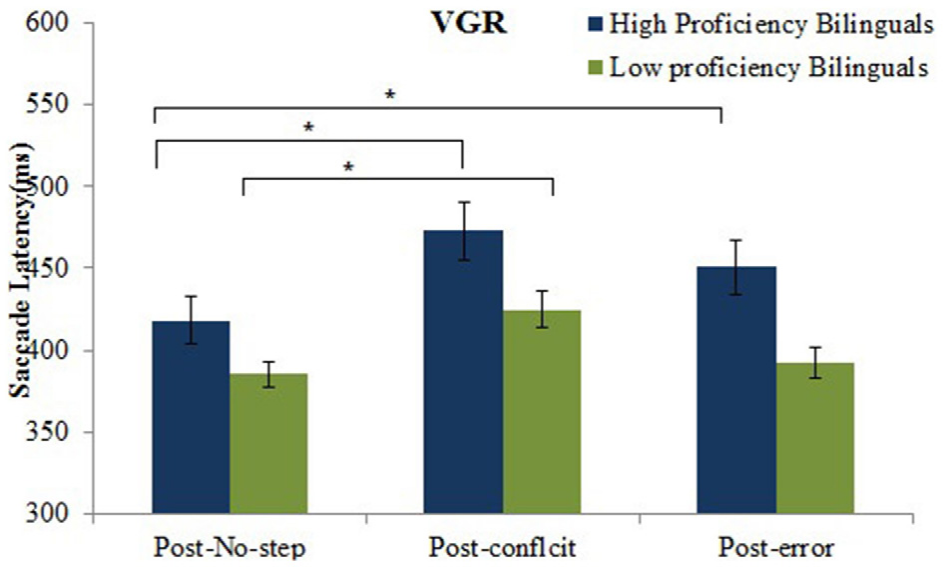
**Mean saccade latencies to the post-conflict, post-error and post-no-step trials for high and low proficient bilinguals in the VGR task**. ^*^p < 0.05.

The results revealed that there was a significant effect of group on the saccade latency for the no-step trials, *F*_(1, 45)_ = 6.9, *p* = 0.01. It showed an overall higher saccade latency for no-step trials for the high proficient bilinguals (447.1 ms, *SE* = 12.8) than the low proficient bilinguals (400.7 ms, *SE* = 12.0). The main effect of the no-step trial type was significant, *F*_(2, 90)_ = 42.5, *p* = 0.001, showing a significantly higher saccade latency for post-conflict trials (448.7 ms, *SD* = 10.35) and post-error trials (421.2 ms, *SE* = 9.4) as compared to the saccade latency for the post-no-step trials (401.7 ms, *SE* = 7.9). The interaction between no-step trial type and group was also significant, *F*_(2, 90)_ = 3.1, *p* = 0.04. *Post-hoc* analysis using Tukey's HSD test, revealed that the high proficient bilinguals showed a significant increase in mean saccade latency for both post-conflict trials (472.6 ms, *SD* = 85.5) and post-error trials (450.5 ms, *SD* = 80.0) as compared to the mean saccade latency on post-no-step trials (418.2 ms, *SD* = 68.3). However, the low proficient bilinguals only showed significant increase in the saccade latency for post-conflict trials (424.9 ms, *SD* = 54.8) as compared to the post no-step trials (385.3 ms, *SD* = 38.7). No significant increase in saccade latency for the post-error trials (391.9 ms, *SD* = 46.9) was observed for the low proficient bilinguals (See Figure 3).

Further, in order to determine the effect of target step delay time on the post-conflict and post-error slowing an additional analysis was conducted. Four conditions, for each post-conflict and post-error trial types, were obtained (i.e., post conflict trial/post-error trials preceded by step trial presented at 50, 100, 150, and 200 ms TSD). The trial presentation was totally random, so the number of post-conflict and post-error trials per TSD was not the same. The average number of post-conflict and post-error trials at each TSD is given in the Table 4 (One subject in the high proficient group did not have any post conflict trial at 50 ms TSD).

**Table 4:** **Mean saccade latency (ms) and number of post-conflict and post-error trials per TSD for both the groups**.

**Group**	**Trial type**	**TSD (ms)**	**Saccade Latency (ms)**	**Avg. no. of trials**
High prof. bilinguals	Post-conflict	50	468.6 (16.9)	22.0 (8.3)
		100	461.0 (16.3)	8.3 (7.4)
		150	463.3 (18.6)	15.5 (8.0)
		200	462.9 (15.4)	14.8 (7.2)
	Post-error	50	438.3 (13.4)	12.6 (8.0)
		100	426.4 (16.7)	16.5 (7.9)
		150	446.5 (15.9)	18.7 (8.1)
		200	445.3 (13.5)	21.3 (7.8)
Low prof. bilinguals	Post-conflict	50	429.6 (15.5)	20.4 (7.1)
		100	423.3 (15.2)	17.0 (7.2)
		150	417.5 (17.0)	15.2 (6.4)
		200	429.8 (14.1)	12.6 (6.3)
	Post-error	50	386.4 (12.3)	14.1 (5.0)
		100	389.3 (15.3)	18.6 (6.6)
		150	396.6 (14.6)	19.2 (6.1)
		200	387.4 (12.3)	23.4 (7.3)

*Standard deviations are given in parentheses*.

A repeated measure ANOVA with post-step trial type (post-conflict, post-error) and step TSD (50, 100, 150, 200 ms) as a within subjects' factor and group (high and low proficient bilinguals) as a between subjects' factor was conducted. The main effect of trial type was found significant, *F*_(1, 44)_ = 29.0, *p* = 0.001, showing higher mean saccade latency for the post-conflict trials (444.5 ms, *SE* = 10.2) than for post-error trials (414.5 ms, *SE* = 8.9). The main effect of group was also significant, *F*_(1, 44)_ = 4.7, *p* = 0.02; showing overall higher post-step slowing for the high proficient bilinguals (451.5 ms, *SE* = 13.5) than for the low proficient bilinguals (407.5 ms, *SE* = 12.4). However, the main effect of step TSD was not significant, *F*_(3, 132)_ = 0.55, *p* = 0.64; showing comparable post-step slowing at all the TSDs[50 ms TSD (430.7 ms, *SE* = 9.2); 100 ms TSD (425.0 ms, *SE* = 10.2); 150 ms TSD (431.0 ms, *SE* = 10.7); and, 200 ms TSD (431.3 ms, *SE* = 9.1)]. The interaction between trial type and group was not significant, *F*_(1, 44)_ = 0.86, *p* = 0.35. Similarly, the interaction between TSD and the group was not significant, *F*_(3, 132)_ = 0.55, *p* = 0.64. The interaction between TSD and trial type was also not found significant, *F*_(3, 132)_ = 0.91, *p* = 0.43. The three way interaction also could not reach the level of significance, *F*_(1, 132)_ = 0.45, *p* = 0.71 (see Table 4).

#### Correlation analysis

In addition to the group comparison, a complementary analysis was done to see how proficiency correlated with the bilinguals' performance on response inhibition measures and performance monitoring. For this purpose, the average scores on subjective and objective proficiency measure were used. At first aggregate of the self-rated proficiency in reading, writing, understanding, and speaking in L2 was obtained followed by calculation of percentage of the same. Then, the average of self-rating and the LexTALE score percentage was calculated. This average proficiency score was used for the correlation analysis.

The correlation analysis showed no significant correlation of TSRT with the L2 proficiency scores, *r*_(47)_ = −0.07, *p* = 0.32. L2 proficiency was negatively correlated with the number of non-canceled saccades at all four TSDs [50 ms TSD, *r*_(47)_ = −0.35, *p* = 0.007; at 100 ms TSD, *r*_(47)_ = −0.32, *p* = 0.01; at 150 ms TSD, *r*_(47)_ = −0.19, *p* = 0.09; and at 200 ms TSD, *r*_(47)_ = −0.32, *p* = 0.01]. The inverse relationship suggests that higher L2 proficiency is associated with a lower number of non-canceled saccades.

**Figure 4:**
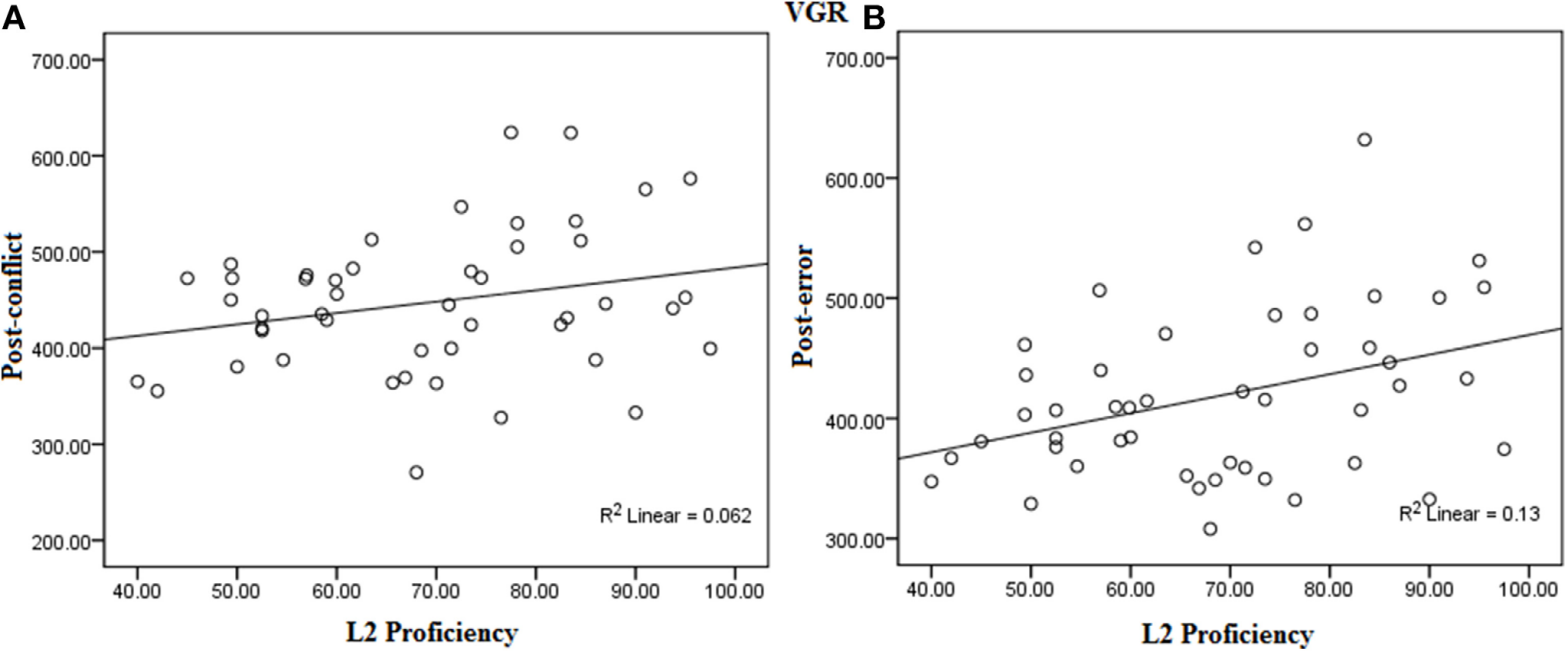
**Correlation between L2 proficiency and (A) Post-conflict slowing and (B) Post-error slowing in the VGR task**.

However, L2 proficiency was positively correlated with the post-step trials. L2 proficiency showed a significant positive correlation with the bilinguals' saccade latencies on the post-conflict trials, *r*_(47)_ = 0.25, *p* = 0.04. Similarly, a positive association of L2 proficiency with post-error slowing, *r*_(47)_ = 0.36, *p* = 0.006; and post-no-step trials, *r*_(47)_ = 0.28, *p* = 0.02, was found. This suggested that the higher L2 proficiency was strongly associated with higher post-step slowing (see Figure 4 for scatter-plots).

### Discussion

The high proficient bilinguals showed significantly greater post-conflict and post-error slowing compared to the post-no-step trials, whereas the low proficient bilinguals only showed post-conflict slowing. The higher post-step slowing in high proficient bilinguals indicates enhanced performance monitoring. The positive correlation between L2 proficiency and post-conflict and post-error slowing, further confirmed the association of higher proficiency with higher performance monitoring. However, no effect of TSD on post-conflict or post-error slowing was observed. No group difference was found for TSRT. The finding on TSRT was further reflected in correlation analysis, showing no association between L2 proficiency and the TSRT, thus, showing no effect of language proficiency on the amount of time taken to cancel the initiated response on the step trials. However, the low proficient group showed a significantly higher number of non-canceled saccades as compared to the high proficient bilinguals. Thus, it shows low proficient bilinguals were poor at redirecting saccades on the step trials as compared to the high proficient bilinguals. It was further corroborated by the significant negative correlation found between L2 proficiency and the number of non-canceled saccades.

## Experiment 2

In Experiment 1, where a robust performance monitoring was observed in high proficient bilinguals as indicated by the elevated saccade latencies for post-conflict and post-error no-step trials, a mixed result for the response inhibition was found. Both, the number of non-canceled saccade and the TSRT are considered good measures of response inhibition, but the two effects were in opposite directions in Experiment 1. The higher number of non-canceled saccades but comparable TSRT for the low proficient bilinguals, as compared to high proficient bilinguals may not suggest enhanced response inhibition in the high proficient bilinguals. We reasoned that the higher number of non-canceled saccades for the low proficient bilinguals could be due to low proficient bilinguals making more reflexive saccades toward the sudden appearance of the target in the VGR task, as compared to the high proficient bilinguals, rather being poor at countermanding the saccade. To test this we used another version of redirect saccade task known as the memory guided redirect task (see Kapoor and Murthy, 2008). In this paradigm, instead of making a saccade directly to the target, the participants are required to memorize the location of the target while looking at the central fixation. After the central fixation disappears, participants need to make a quick saccade to the target location (on the no-step trials) and to stop and redirect gaze to the second target on the step-trials. So, the participants were required to refrain from making a saccade to the target until cued to go.

We predicted that the high proficient bilinguals should show higher post-conflict and post-error slowing (as in the Experiment 1), as compared to low proficient bilinguals because of their enhanced performance monitoring. Secondly, if poor response inhibition in low proficient bilinguals was the reason for the higher number of non-canceled saccades in VGR task, then in the MGR task as well, low proficient bilinguals would continue to show a higher number of non-canceled saccades compared to the high proficient bilinguals. However, the difference between the two groups for the number of non-canceled saccades would be eliminated in MGR if it was a consequence of low proficient bilinguals making more reflexive saccades toward the target in the VGR version of the task.

### Methods

#### Participants

Another group of high (*N* = 18) and low (*N* = 20) proficient Hind-English bilinguals were recruited in the second study, using the same criteria as in Experiment 1. All the participants were administered the same language background and proficiency measures (see Tables 5, 6). All the participants gave their informed consent prior to participation in the study and were paid for their participation.

**Table 5:** **Self-ratings for reading, writing, speaking, and comprehension in L1 and L2**.

	**Speaking**		**Listening**		**Reading**		**Writing**	
	**LI**	**L2**	**LI**	**L2**	**LI**	**L2**	**LI**	**L2**
HPB	4.8 (0.38)	4.2 (0.73)	4.8 (0.32)	4.6 (0.50)	4.7 (0.46)	4.6 (0.48)	4.2 (0.75)	4.2 (0.75)
LPB	4.6 (0.50)	2.5 (0.94)	4.6 (0.48)	2.8 (0.98)	4.8 (0.36)	3.4 (0.99)	4.5 (0.51)	3.1 (0.81)
		^**^		^**^		^**^		^**^

*Standard deviations are given in parentheses*.

*L1, Hindi; L2, English. High and low proficiency based on L2 proficiency*.

** *p < 0.01*.

**Table 6:** **Demographic details, non-verbal IQ, LexTALE, and comprehension passage scores of high-proficient bilinguals (HPB) and low proficient bilinguals (LBP)**.

	**HPB**	**LPB**
Mean age	22.1 (2.9)	20.4 (2.2)
Mean formal age of L1 acquisition (years)	3.5 (0.51)	3.8 (0.52)
Mean formal age of L2 acquisition (years)	3.7 (0.64)	4.1 (0.71)
Hours of work related activity in L1	3.1 (0.96)	4.5 (0.99)^**^
Hours of work related activity in L2	4.0 (1.4)	0.92 (0.71)^**^
Non-verbal IQ	53.2 (0.39)	51.6 (0.40)
Socio-economic status	2.3 (0.50)	2.2 (0.44)
Mean score in L1 comprehension (out of 5	4.4 (0.78)	4.6 (0.48)
Mean score in L2 comprehension (out of 5)	4.2 (0.82)	1.9 (1.0)^**^
LexTALE score percentage	85.3 (7.0)	58.1 (8.3)^**^

Standard deviations are given in parentheses

*L1, Hindi; L2, English. Non-verbal IQ: Ravens Progressive Matrices, out of 60. See text for explanation*.

** *p < 0.01*.

#### Procedure

**Figure 5:**
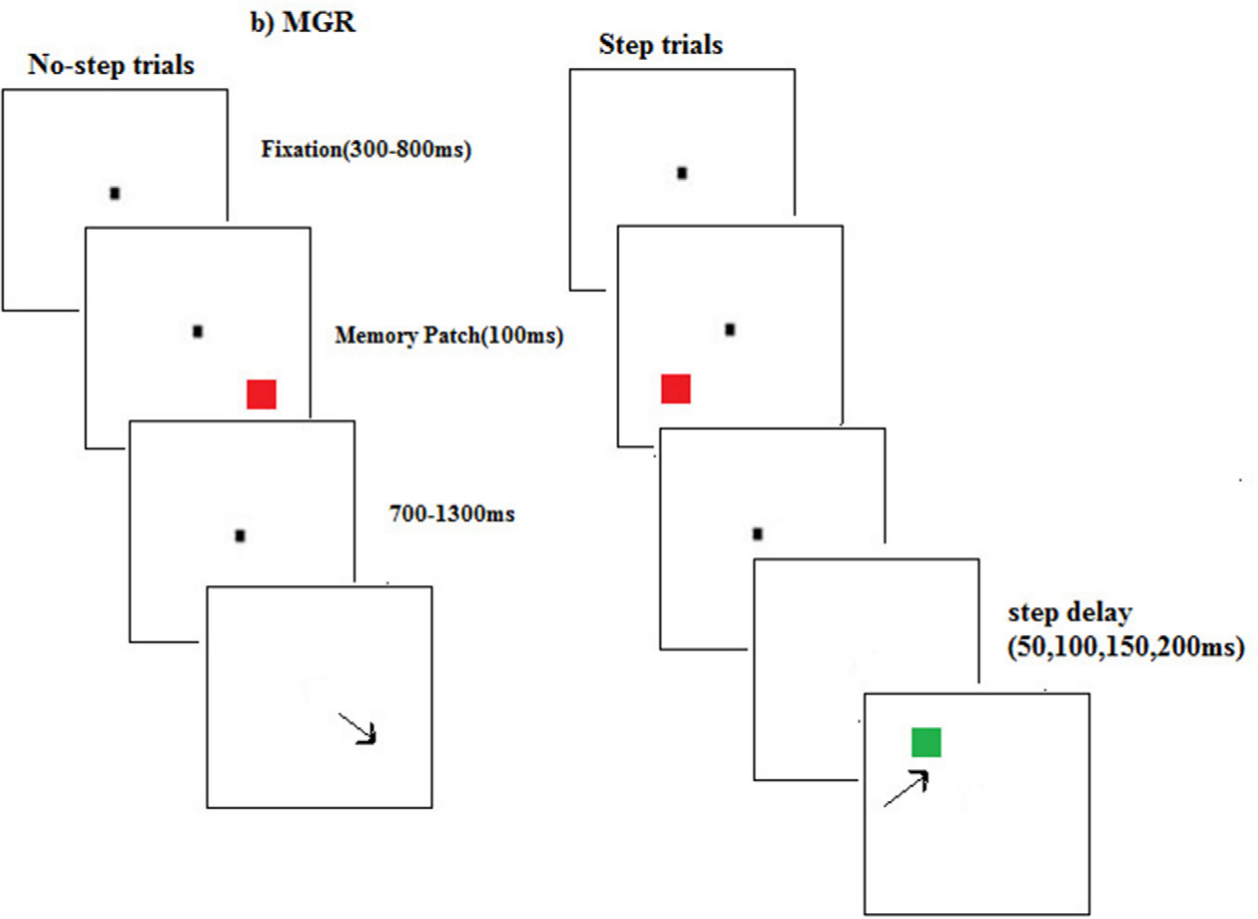
**A sample trial sequence for step and no-step trials in the memory guided redirect (MGR) saccade task**. In no-step trials participants were required to make saccade to the remembered target location (red square). In the step trial participants were instructed to redirect gaze to the second target (green square) instead of looking at the remembered location of the first target.

The experiment began with a calibration at 13 points. The proportion of step and no-step trials was same as in Experiment 1. A central fixation was presented for a variable period of time ranging from 300 to 800 ms. While participants were looking at the central fixation, a target appeared at one of the six possible locations (as in Experiment 1) and disappeared after 100 ms. After a variable period of 700–1300 ms from the offset of the target, the central fixation disappeared signaling to make a saccade to the target location on no-step trials. However, on the step trials, the second target appeared after the target step delay (of 50, 100, 150, or 200 ms) with the fixation offset. On these trials, participants were required to cancel the saccade to the memorized location of the first target and to redirect gaze to the new target. The experiment had a total of 600 trials, out of which 240 were step trials (with 60 trials presented at each TSD) (See Figure 5).

### Results

#### Number of non-canceled saccades

The main effect of group was not found significant, *F*_(1, 36)_ = 2.2, *p* = 0.14, indicating comparable number of non-canceled saccades for the high (18.7, *SE* = 1.8) and the low proficient bilinguals (22.5, *SE* = 1.7). The main effect of TSD was significant, *F*_(3, 108)_ = 22.5, *p* = 0.001, revealing a significantly higher number of non-canceled saccades with an increase in the TSD. The interaction between the group and the TSD for non-canceled saccades did not reach levels of significance, *F*_(3, 108)_ = 0.68, *p* = 0.56 (See Table 7).

**Table 7:** **Mean number of non-canceled saccades per target step delay (TSD) for the groups**.

	**Number of non-canceled saccades (MGR)**	
**TSD (ms)**	**High proficient bilinguals**	**Low proficient bilinguals**
50	13.2 (2.4)	18.0 (2.3)
100	16.2 (2.1)	20.5 (2.0)
150	20.4 (1.8)	25.7 (1.7)
200	23.8 (2.2)	26.9 (2.1)

*Standard deviations are given in parentheses*.

#### Trial switch reaction time (TSRT)

**Figure 6:**
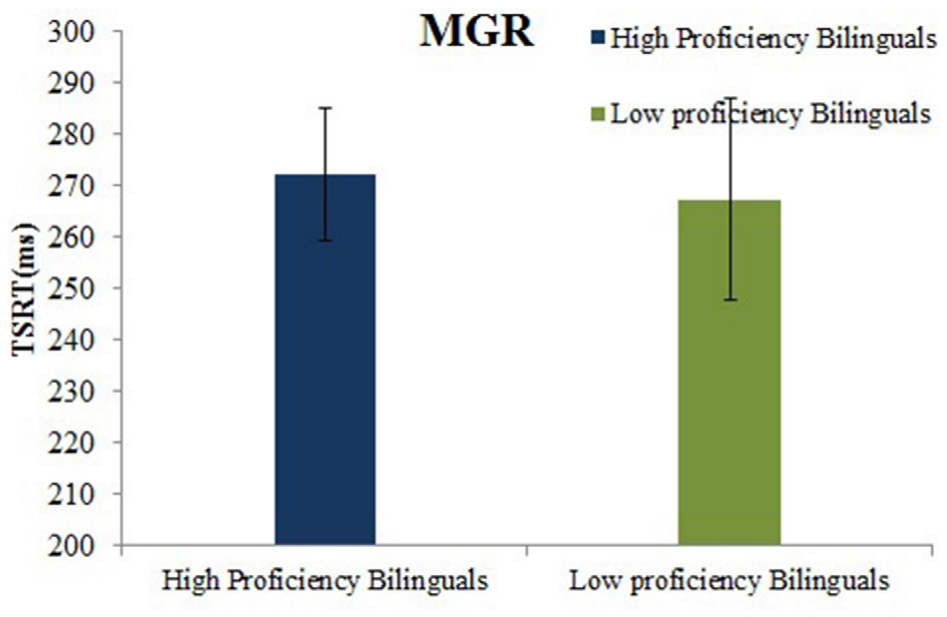
**Mean TSRT (ms) for high and low proficient bilinguals on the MGR task**.

The high and low proficient bilinguals did not differ on TSRT, *t*_(36)_ = 0.20, *p* = 0.84. Both the high (272.1 ms, *SD* = 53.4) and low proficient bilinguals (267.2 ms, *SD* = 87.9) took a similar amount of time to inhibit the saccade to the first target (see Figure 6).

#### Post-error and -post conflict slowing (performance monitoring):

Saccade latencies less than 80 ms (anticipatory) and greater than 1000 ms were excluded from the analysis. The main effect of group was significant, *F*_(1, 36)_ = 8.8, *p* = 0.005, suggesting that high proficient bilinguals (513.6 ms, *SE* = 12.8) showed a higher post-step slowing than the low proficient bilinguals (463.0 ms, *SE* = 11.7) on all the no-step trials. The main effect of no-step trial type was also significant, *F*_(2, 72)_ = 4.5, *p* = 0.01, showing an increase in the saccade latency for post-conflict trials (498.7 ms, *SE* = 8.7) as compared to the post-no-step trials (479.6 ms, *SE* = 9.1).

**Figure 7:**
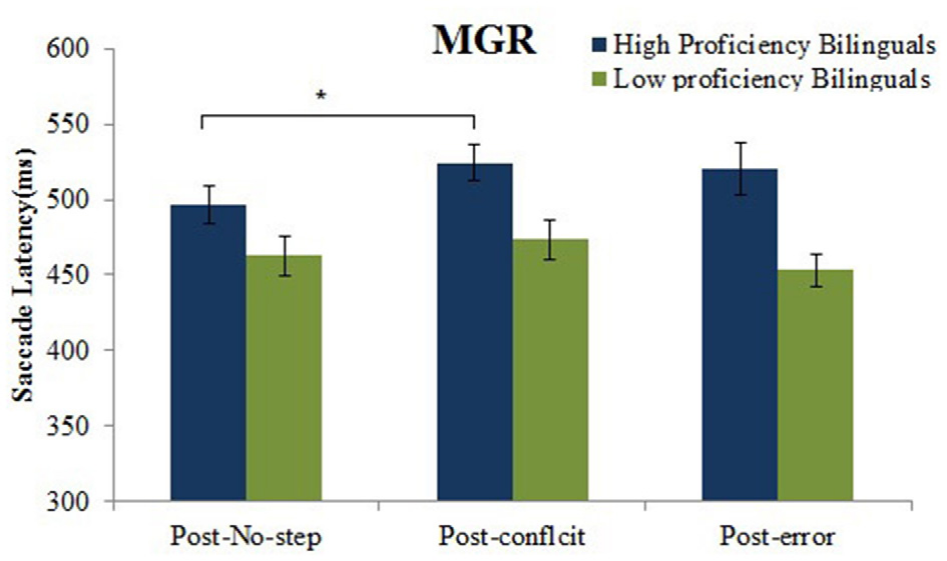
**Mean saccade latencies to the post-conflict, post-error and post-no-step trials for high and low proficient bilinguals in the MGR task**. ^*^p < 0.05.

The interaction between group and no-step trial type was significant, *F*_(2, 72)_ = 3.5, *p* = 0.04. Further analysis using the *post-hoc* using Tukey's HSD revealed that for the high proficient bilinguals the mean post-error saccade latency (520.3 ms, *SE* = 74.3) did not differ significantly from the mean post-no-step saccade latency (496.6 ms, *SE* = 53.5). However, the mean post-conflict saccade latency (524.0 ms, *SE* = 50.4) was significantly higher than the mean post-no-step saccade latency. For the low proficient bilinguals the *post-hoc* analysis revealed no significant difference in the mean post-conflict (473.3 ms, *SE* = 57.0) or mean post-error saccade latency (453.1 ms, *SE* = 46.5) as compared to the mean post-no-step saccade latency (462.6ms, *SE* = 12.5) (See Figure 7).

As in Experiment 1, an additional analysis was performed to examine the effect of target step delay time on the post-conflict and post- error slowing for both the groups. The trial presentation was totally random, so the number of post-conflict and post-error trials per TSD was not same. The average number of post-conflict and post-error trials per TSD is given in the (See Table 8) (Two high proficient bilinguals and one low proficient bilingual did not have any post-error trial at 50 ms TSD).

**Table 8:** **Mean saccade latency (ms) and number of post-conflict and post-error trials per TSD for both the groups**.

**Group**	**Trial type**	**TSD (ms)**	**Saccade Latency (ms)**	**Avg. no. of trials**
High prof. bilinguals	Post-conflict	50	529.3 (17.2)	29.2 (5.6)
		100	509.3 (20.8)	25.4 (4.4)
		150	524.5 (20.3)	22.7 (4.9)
		200	511.9 (15.2)	20.9 (6.1)
	Post-error	50	490.8 (24.8)	7.4 (5.2)
		100	505.3 (21.0)	10.1 (4.3)
		150	532.5 (18.8)	12.2 (5.1)
		200	492.0 (17.3)	14.9 (5.3)
Low prof. bilinguals	Post-conflict	50	488.7 (15.2)	24.0 (7.5)
		100	452.2 (18.5)	22.7 (7.6)
		150	473.3 (18.1)	19.7 (5.4)
		200	467.5 (13.5)	18.0 (6.9)
	Post-error	50	452.9 (22.0)	10.3 (6.9)
		100	459.4 (18.7)	13.2 (7.9)
		150	439.7 (16.7)	14.9 (6.4)
		200	477.0 (15.4)	16.8 (6.7)

*Standard deviations are given in parentheses*.

A repeated measure ANOVA showed that the main effect of trial type was not significant, *F*_(1, 32)_ = 2.9, *p* = 0.09, showing comparable saccade latencies for post-conflict (494.6 ms, *SE* = 9.1) and post error trials (481 ms, *SE* = 10.3). The main effect of group was found highly significant, *F*_(1, 32)_ = 7.2, *p* = 0.01, showing higher post-step slowing for high proficient bilinguals (511.9 ms, *SE* = 13.4) compared to the low proficient bilinguals (463.8 ms, *SE* = 11.9). However, the main effect of TSD was not found significant, *F*_(2, 96)_ = 0.46, *p* = 0.70; showing comparable saccade latencies for post-step trials at all the TSDs [490.4 ms, *SE* = 13.0) at 50 ms TSD; 481.5 ms (*SE* = 10.9) at 100 ms TSD; 492.5 ms (*SE* = 10.8) at 150 ms TSD; and 487.1 ms (*SE* = 7.6) at 200 ms TSD].

The two way interaction between trial type and group was not found significant, *F*_(1, 32)_ = 0.00, *p* = 0.97. Similarly, the TSD x group interaction was not significant, *F*_(3, 96)_ = 1.7, *p* = 0.17. The interaction between trial type and TSD also did not reach the level of significance, *F*_(3, 96)_ = 1.2, *p* = 0.20. The three way interaction of group × TSD × trial type was not found significant, *F*_(3, 96)_ = 1.0, *p* = 0.38.

#### Correlation analysis

The same procedure to calculate average L2 proficiency score, for correlation analysis, used in experiment 1, was performed. No significant correlation between L2 proficiency and TSRT was found, *r*_(38)_ = −0.05, *p* = 0.37. The L2 proficiency showed negative correlation with the number of non-canceled saccades for all the four TSDs [50 ms TSD, *r*_(38)_ = −0.32, *p* = 0.02; 100 ms TSD, *r*_(38)_ = −0.25, *p* = 0.05; 150 ms TSD, *r*_(38)_ = −0.40, *p* = 0.006; and 200 ms TSD, *r*_(38)_ = −0.07, *p* = 0.33] suggesting higher L2 proficiency was associated with a lower number of non-canceled saccades in bilinguals.

**Figure 8:**
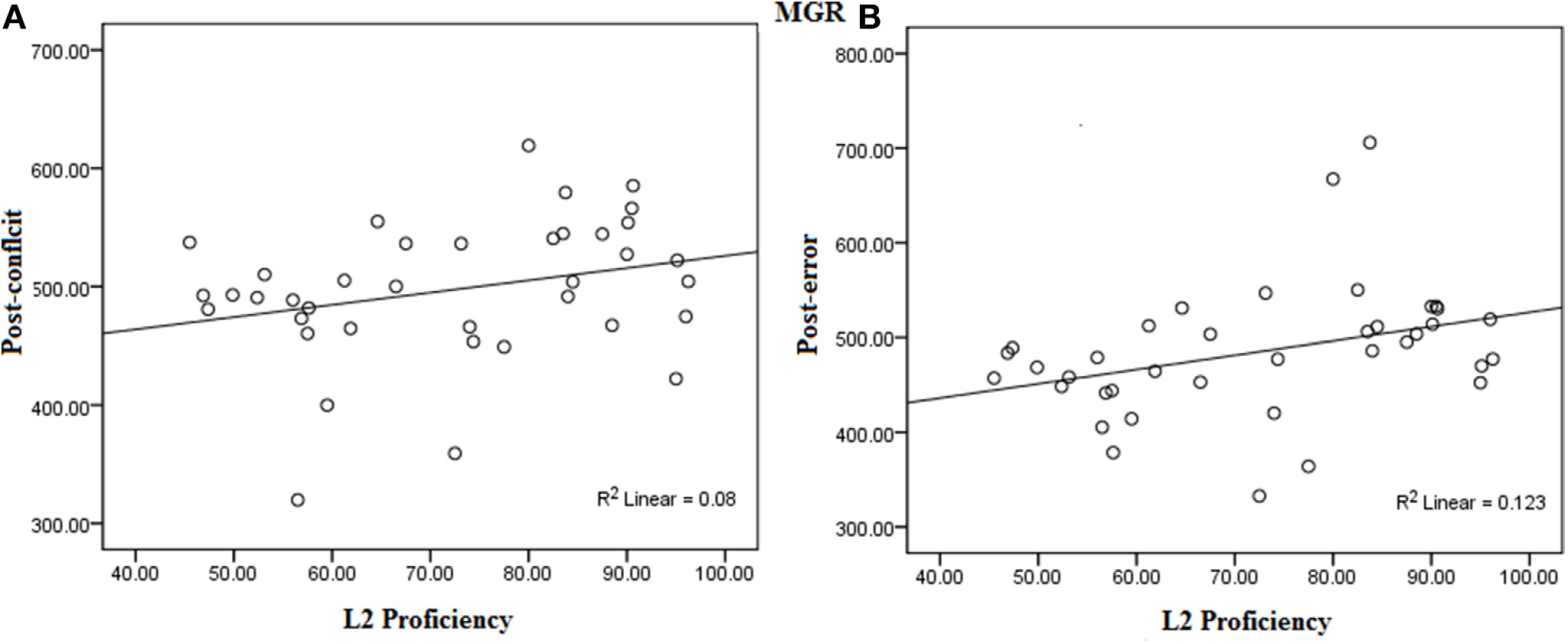
**Correlation between L2 proficiency and (A) Post-conflict slowing and (B) Post-error slowing in the MGR task**.

The L2 proficiency was found to be positively correlated with the saccade latency on the post-conflict trials, *r*_(38)_ = 0.28, *p* = 0.04; and post-error trials, *r*_(38)_ = 0.35, *p* = 0.01. Thus showing that, higher proficiency was strongly associated with higher post-no-step trials. However, no significant correlation between L2 proficiency and post-no-step trials was found, *r*_(38)_ = 0.16, *p* = 0.16 (see Figure 8 for scatter-plots).

### Discussion

The results suggest greater post-step slowing for the high proficient bilinguals as compared to the low proficient bilinguals. It was found that the high proficient bilinguals showed significantly greater post-conflict, whereas no such slowing was observed for the low proficient bilinguals. The positive correlation between L2 proficiency and post-conflict and post-error trials further provided evidence for the enhanced performance monitoring for high proficient bilinguals. Similar to Experiment 1, no effect of TSD on post-conflict or post-error slowing was found.

Interestingly, in this study, the previously observed advantage on the number of non-canceled saccades on the step trials for the high proficient bilinguals was not found in the group-wise analysis. However, correlation analysis showed a significant negative association between L2 proficiency and the non-canceled saccades at various TSDs. No effect of L2 proficiency was found for TSRT, in both group-wise and correlation analysis.

## General discussion

Recent studies have shown enhanced monitoring system in bilinguals compared to monolinguals (Costa et al., 2009), as well as high proficient bilinguals to low proficient bilinguals (Singh and Mishra, 2013). We aimed not only to extend these findings, but also to explore other facets of monitoring such as performance monitoring in bilinguals. We examined whether response inhibition and performance monitoring within the oculomotor domain are affected by language proficiency in bilinguals. To this end, we compared high and low proficient bilinguals on two versions of the oculomotor redirect task. Using switch reaction time (TSRT) and a number of non-canceled saccades as a measure of response inhibition and post-step slowing as a measure of performance monitoring, we observed two important results. First, as per our predictions, the high proficient bilinguals showed greater post-step slowing as compared to the low proficient bilinguals, on both the VGR and MGR tasks. Secondly, the high and low proficient bilinguals exhibited comparable TSRT, on both VGR and MGR tasks. However, the low proficient bilinguals exhibited a higher number of non-canceled saccades as compared to the high proficient bilinguals but only on the VGR task. The implications of the results are discussed in the following sections.

### Performance monitoring

Our results revealed that the high proficient bilinguals showed more post-stop slowing compared to the low proficient bilinguals, which we take as evidence for performance monitoring. This pattern was observed in both the versions of the redirect task in our study. This finding is in compliance with other (monolingual) saccadic countermanding studies showing post-stop/step slowing as a consequence of performance monitoring (Cabel et al., 2000; Kornylo et al., 2003; Farooqui et al., 2011). As expected, our results clearly show modulatory effects of language proficiency on performance monitoring in bilinguals. Confirming this interpretation, a correlation analysis showed a positive relation between L2 proficiency and post-conflict and post-error slowing, indicating higher performance monitoring for bilingual participants with higher L2 proficiency. Thus, our findings support and strengthen the previous findings, which suggest that bilingualism leads to enhanced monitoring (Costa et al., 2008, 2009) and is modulated by higher L2 proficiency (Singh and Mishra, 2013). Festman and Münte (2012) had observed that non-switchers were better at self-monitoring and also displayed low error related negativity. In this study, participants were divided into groups of low and high switchers and switching was linked to cognitive control. In our study we did not group participants with regard to their switching pattern but with proficiency. Therefore, it is possible that apart from proficiency, “switching” behavior of participants could have played a role in enhancing their monitoring. However, there are important differences in these studies. Festman and Münte (2012) had used Wisconsin Card Sorting Test and a Flanker task, a task that involves conflict. We used tasks that called for change in the action plan under uncertain situations. Thus, it is quite possible that the performance monitoring ability seen in bilinguals could relate to attributes like proficiency or switching and also manifest differently in different tasks. Recently, Valian (2014) has argued that the effects seen on tasks in bilingual cognitive control studies are often related to specific task demands and how these tasks mimic the bilingual behavior.

The group-wise analysis of post-conflict and post-error slowing also suggest involvement of top down control. An interesting pattern emerged when post-error and post-conflict trials were compared with post-no-step trials. It was found that the high proficient bilinguals showed robust slowing down on post-conflict as well as post-error trials on the VGR task, whereas only showed post-conflict slowing on the MGR task. The low proficient bilinguals showed no difference in the saccade latency on the post-error and the post-conflict trials when compared with the post-no-step trials for the MGR task, but showed post-conflict slowing when compared with the post-no-step trials for the VGR task. This shows that the high proficient bilinguals relied more on the detection of conflict for performance adjustment. However, dependence on the error detection, for performance monitoring in high proficient bilinguals seemed to vary with the task. Our findings are in consonance with several studies where post-conflict (Verbruggen and Logan, 2008; Bissett and Logan, 2011; Stuphorn and Emeric, 2012) and post-error (Rabbitt, 1966; Spunt et al., 2012; also see Ridderinkhof et al., 2004) slowing has been observed. Our data suggest that high proficient bilinguals are better at evaluating performance and making appropriate changes to response strategies as compared to the low proficient bilinguals.

Further, to see the effect of target step delay on the post-step trials, saccade latencies for post-conflict and post-error trials per TSD were compared. Previous studies with countermanding tasks have shown that canceling or redirecting of saccades on the step-trials at shorter TSDs is easier as compared to the step-trials at longer TSDs which require higher inhibition (Logan and Cowan, 1984). In the present study, it was expected that longer TSDs would result into more post-conflict and post-error slowing. Contrary to this, it was found that short and long TSD had no effect on the post-conflict or post-error slowing for both the groups in the two experiments. We assume large variation in the frequency of occurrence of number of post-conflict and post-error trials per TSD (See Tables 4, 8) may have resulted in the absence of any effect of TSD on the post-step trials.

In sum, our results show modulation of performance monitoring by L2 proficiency. This shows that high proficient bilinguals are more flexible in behavioral adjustments compared to low proficient bilinguals. This finding goes in line with the studies where positive consequences of language proficiency in bilinguals have been reported (Tao et al., 2011; Coderre et al., 2012; Singh and Mishra, 2012, 2013; Khare et al., 2013).

### Response inhibition

The TSRT and number of non-canceled saccades were used as a measure of response inhibition in the present study. Both the groups showed increase in TSRT at longer target step delay, but no group difference was found for TSRT. Our results showed that both high and low proficient bilinguals exhibited an equivalent amount of TSRT in both the versions of the redirect task. This observation was further confirmed by correlation analysis showing no significant correlation between L2 proficiency and TSRT, thus, suggesting no effect of L2 proficiency on the amount of time taken to inhibit the planned saccade on the step trials.

Contrary to the findings on TSRT, a group difference was found for another measure of response inhibition, i.e., number of non-canceled saccades, in the VGR task. The low proficient bilinguals showed a higher number of non-canceled saccades than the high proficient bilinguals in the VGR task. Furthermore, a significant negative correlation between L2 proficiency and the number of non-canceled saccades was also found, indicating a lower number of non-canceled saccades on the step-trials for those with higher L2 proficiency.

The equivocal findings on the effect of L2 proficiency, on the two measures of response inhibition, from the VGR task made it difficult to conclude about response inhibition in bilinguals. We assumed there may be a possibility that low proficient bilinguals were more prone to make reflexive saccades toward the exogenously appearing target in the VGR task, thus leading to higher number of non-canceled saccades, as compared to the high proficient bilinguals. To this end, we used the MGR task in the Experiment 2, which required participants to program saccades toward the memorized target location instead of the target itself. The result from the group comparison showed no difference in the number of non-canceled saccades for both high and low proficient bilinguals in MGR task. This indicated that the difference in the number of non-canceled saccades for the two language groups in the VGR task was not due to enhanced response inhibition in high proficient bilinguals, but due to low proficient bilinguals making more reflexive saccades toward the target. However, for the same task, correlation analysis showed a significant negative association between L2 proficiency and the number of non-canceled saccades.

Thus, the overall pattern of results suggests a limited advantage on response inhibition for the high proficient bilinguals. This pattern is consistent with previous findings where bilinguals did not show any advantage over monolinguals when tested on manual response inhibition tasks such as the Stop-Signal task and Go/No-Go task (Colzato et al., 2008; Martin-Rhee and Bialystok, 2008; Hilchey and Klein, 2011; Morales et al., 2013).

Our previous studies comparing high and low proficient bilinguals (Singh and Mishra, 2012, 2013) used a saccadic Stroop task and found evidence for inhibition as well as monitoring, which is in contradiction with the results of the present study. However, it is important to note that the Stroop and redirect tasks tap different forms of inhibitory control (Nigg, 2000). In the Stroop task there was no uncertainty with regard to saccade preparation time or cancelation of planned saccades. The results from the present study support the proposal that bilinguals are better at tasks that offer conflict at the perceptual level like the Stroop task, rather than on tasks that offer inhibition of response, as in stop-signal or redirect task (Blumenfeld and Marian, 2014). Our findings cohere with the emerging view that bilinguals show an advantage on only certain forms of inhibition (Luk et al., 2010; Esposito et al., 2013).

These results should be viewed while keeping several contingent factors that might affect bilingual's performances on attention tasks. The bilinguals used in our study came from a student population who had Hindi as their dominant language. However, they differed in their English proficiency. It is important to note that the overall language use and proficiency may differ dynamically across India depending on several socio-linguistic factors (Pattanayak, 1991). Moreover, language use may also differ depending on the place of use i.e., work vs. home. Therefore, while we conclude that overall language proficiency may have substantial effects on executive control performances; future studies should consider such socio-linguistic variables while comparing bilinguals. This study also demonstrates that the influence of bilingualism on cognitive and attention control may depend a lot on the tasks, and their demands. Our study has a limitation since we did not control the participants for their capacity in working memory or visual short term memory. It is likely that individual differences affect performance on tasks that call for dynamic oculomotor control. This should be controlled for in future research.

In summary, the present study demonstrates that language proficiency modulates performance monitoring in bilinguals. Thus, indicating a higher cognitive flexibility and a superior ability to adjust behavior in the high proficient bilinguals, this facilitates attainment of the cognitive goal.

### Conflict of interest statement

The authors declare that the research was conducted in the absence of any commercial or financial relationships that could be construed as a potential conflict of interest.
